# Calcium Signaling in T Cells Is Induced by Binding to Nickel-Chelating Lipids in Supported Lipid Bilayers

**DOI:** 10.3389/fphys.2020.613367

**Published:** 2021-01-21

**Authors:** Tommy Dam, Victoria Junghans, Jane Humphrey, Manto Chouliara, Peter Jönsson

**Affiliations:** ^1^Department of Chemistry, Lund University, Lund, Sweden; ^2^Department of Chemistry, University of Cambridge, Cambridge, United Kingdom

**Keywords:** calcium signal, T-cell receptor, CD45, kinetic segregation model, CD2, ligand-independent activation

## Abstract

Supported lipid bilayers (SLBs) are one of the most common cell-membrane model systems to study cell-cell interactions. Nickel-chelating lipids are frequently used to functionalize the SLB with polyhistidine-tagged ligands. We show here that these lipids by themselves can induce calcium signaling in T cells, also when having protein ligands on the SLB. This is important to avoid “false” signaling events in cell studies with SLBs, but also to better understand the molecular mechanisms involved in T-cell signaling. Jurkat T cells transfected with the non-signaling molecule rat CD48 were found to bind to ligand-free SLBs containing ≥2 wt% nickel-chelating lipids upon which calcium signaling was induced. This signaling fraction steadily increased from 24 to 60% when increasing the amount of nickel-chelating lipids from 2 to 10 wt%. Both the signaling fraction and signaling time did not change significantly compared to ligand-free SLBs when adding the CD48-ligand rat CD2 to the SLB. Blocking the SLB with bovine serum albumin reduced the signaling fraction to 11%, while preserving CD2 binding and the exclusion of the phosphatase CD45 from the cell-SLB contacts. Thus, CD45 exclusion alone was not sufficient to result in calcium signaling. In addition, more cells signaled on ligand-free SLBs with copper-chelating lipids instead of nickel-chelating lipids and the signaling was found to be predominantly via T-cell receptor (TCR) triggering. Hence, it is possible that the nickel-chelating lipids act as ligands to the cell’s TCRs, an interaction that needs to be blocked to avoid unwanted cell activation.

## Introduction

T-cell activation is initiated by the binding of T-cell receptors (TCRs) to their cognate antigen on a meeting cell. This results in phosphorylation of cytoplasmic immunoreceptor tyrosine-based activation motifs (ITAMs) in the TCR complex, which starts a cascade of chemical reactions resulting in the release of calcium from endoplasmic reticulum Ca^2+^ stores (Chakraborty and Weiss, 2014). The depleted Ca^2+^ stores in turn activate different Ca^2+^ release-activated calcium channels in the plasma membrane to sustain the Ca^2+^ level, ultimately resulting in cytokine production and cell activation (Lewis, 2001; Oh-hora and Rao, 2008). In a resting T cell the ITAMs are kept unphosphorylated by the phosphatase CD45, which according to the kinetic segregation mechanism is excluded due to its large size from the close contacts formed when the TCR binds antigen (Davis and van der Merwe, 2006). TCR triggering and subsequent calcium signaling has traditionally been studied *in vitro* by ligating the TCR to antibodies coated on a glass slide, but has also been achieved using supported lipid bilayers (SLBs) containing peptide-presenting major histocompatibility complex molecules specific for the TCR (Grakoui et al., 1999; Huppa et al., 2010; O’Donoghue et al., 2013). However, recent studies have shown that T-cell signaling can also be induced without directly binding to the TCR (Chang et al., 2016; Ponjavic et al., 2018; Santos et al., 2018; Fernandes et al., 2019). The mechanism for this ligand-independent triggering is due to exclusion of CD45 from the closed contacts formed between the T cell and a functionalized surface. If the area of the close contact is sufficiently large then the TCR will have time to be net phosphorylated and triggered before it diffuses out of the contact (Davis and van der Merwe, 2006).

Supported lipid bilayers are one of the most common membrane model systems and have been used extensively to study cell-cell interactions (Castellana and Cremer, 2006; Dustin, 2009; Jönsson et al., 2016; Biswas and Groves, 2019; Jenkins et al., 2019) including ligand-independent triggering (Chang et al., 2016; Fernandes et al., 2019). Protein ligands are typically anchored to the SLB via polyhistidine-tags that bind to nickel-chelating lipids [DGS-NTA(Ni)] in the SLB (Nye and Groves, 2008). However, ligand-free SLBs containing 5% DGS-NTA(Ni) have previously been observed to bind Jurkat T cells and even induce calcium signaling in the cells (Ponjavic et al., 2018). SLBs will generally not be ligand-free, but instead functionalized with protein ligands that could shield the influence of the nickel-chelating lipids on the cell. The aim of this study was to investigate how DGS-NTA(Ni) influences calcium signaling when there are also receptor-binding ligands on the SLB and to better understand the mechanism behind this. This is important to understand how to keep SLB-binding cells in a resting state and to avoid unwanted cell signaling, but also to utilize this phenomenon as an artificial means to study the molecular mechanisms of T-cell signaling.

As a model system we used Jurkat T cells expressing non-signaling rat CD48 that binds fluorescently labeled rat CD2, which was anchored to an SLB at different concentrations. The rat CD2-CD48 (CD2-CD58 in humans) interaction is argued to align immune-cell surfaces *in vivo* to facilitate the interaction between TCRs and antigen (James and Vale, 2012; Jönsson et al., 2016). It also creates a ∼15 nm cell gap that effectively excludes CD45, an event that by itself can induce T-cell signaling by ligand-independent triggering (Chang et al., 2016; Fernandes et al., 2019). The cells were loaded with a calcium-sensitive dye that upon calcium signaling in the cells gave rise to a sharp increase in intensity. The cells were added to SLB systems with different amounts of DGS-NTA(Ni) and CD2 densities and both the fraction of cells that signaled as well as the time between cell binding and signaling was monitored. It was found that DGS-NTA(Ni) can significantly induce calcium signaling in the cells even when having CD2 in the SLB. It was furthermore demonstrated that DGS-NTA(Ni) dominated over signaling caused by ligand-independent triggering due to CD2 binding CD48 for the current system, and the fraction of signaling cells decreased to 11% when blocking the SLB with bovine serum albumin (BSA) before adding the cells. The signaling was also found to be predominantly via TCR triggering since TCR-deficient J.RT3-T3.5 cells signaled significantly less on DGS-NTA(Ni). To further investigate the mechanism by which DGS-NTA(Ni) induces calcium signaling we labeled the cells with fluorescent antibodies against CD45 and monitored how these molecules distributed in the cell-SLB contact of the different SLBs. CD45 was observed to be excluded from the cell-SLB contacts at a similar level for all CD2-containing systems, independent of the signaling fraction, indicating that the main mechanism for calcium signaling induced by DGS-NTA(Ni) in this study is not ligand-independent triggering but could instead be due to TCR binding.

## Method

### Cell Lines, Culture and Flow Cytometry

E6.1 Jurkat T cells (ATCC) expressing non-signaling rat CD48 and human leukocyte antigen DQ8-glia-α1 (HLA-DQ8-glia-α1) were made as described by Junghans et al. (2020). The cells were kept in RPMI 1640 medium (Sigma-Aldrich), which was supplemented with 10% fetal bovine serum (FBS; Sigma-Aldrich), 2% L-glutamine (Sigma-Aldrich), 1% sodium pyruvate (Sigma-Aldrich), 1% HEPES (Sigma-Aldrich), and 1% Penicillin-Streptomycin (Sigma-Aldrich). The Jurkat T cells were cultured at 37°C and 5% CO_2_ and had a concentration of 5 × 10^5^ cells/ml on the day of the experiment. THP-1 cells and J.RT3-T3.5 cells (ATCC) were cultured using the same conditions and supplemented cell medium as described above.

The amount of CD48, TCR, and CD45 on the cells was determined using flow cytometry and Quantibrite analysis (BD Biosciences). 0.5 × 10^6^ Jurkat T cells were centrifuged for 3 min at 1,200 rpm and washed twice with phosphate-buffered saline (PBS, Merck) containing 0.05% sodium azide. The cells were labeled for 45 min at 4°C with isotype phycoerythrin (PE) α-mouse IgG1 (clone MOPC-21, #400112, BioLegend, 1:10 dilution), PE α-rat CD48 (clone OX-45, #MA5-17528, Thermo Fisher Scientific; 1:10 dilution), PE α-human CD45 (clone 2D1, #368509, BioLegend, 1:10 dilution) and PE α-human CD3 (clone OKT3, #317307, BioLegend, 1:10 dilution) and washed twice with PBS + 0.05% sodium azide before analysis in a BD Accuri C6 Flow Cytometer (BD Biosciences). BD Quantibrite PE beads (#340495, BD Biosciences) were used for quantification purposes and measured alongside the antibody-stained cells. This allowed for the calculation of the total number of antibodies per cell, which, at saturating concentrations of antibodies, was assumed to be equal to the total number of receptors per cell (Poncelet and Carayon, 1985). With the OKT3 antibody targeting CD3ε the TCR number per cell was obtained by dividing the CD3 count by two. All data were analyzed using FlowJo (v10.5.2, BD Biosciences) and Microsoft Excel (Microsoft).

### Calcium Imaging and Antibody Labeling

The cells were prepared for calcium imaging following the protocol outlined by Santos et al. (2018) with minor modifications. Approximately 1 × 10^6^ Jurkat cells were loaded with 1 μl of a 1 mM Fluo4-AM solution (#F-14217, Invitrogen) with 100 μl of supplement-free RPMI 1640 medium (Sigma-Aldrich) and 100 μl HEPES buffer saline solution (HBS; 10 mM HEPES, 150 mM NaCl, pH 7.4) supplemented with 2.5 mM probenecid (AAT Bioquest). The cell solution was incubated for 10 min at 37°C followed by a 20 min incubation at room temperature. The cells were washed three times with HBS buffer + 2.5 mM probenecid before being resuspended in 200 μl of HBS-probenecid buffer solution. 15 μl of the Fluo4-AM-loaded cell solution was added to different surfaces enclosed by a press-to-seal silicon well (4.5 mm in diameter, 1.6 mm in depth, Grace Bio-Labs).

To measure the distribution of CD45 and TCR in the cell-SLB contacts 0.25 × 10^6^ Jurkat T cells were labeled either with 6 μg/ml of Alexa Fluor 488 conjugated anti-CD45 antibodies (clone HI30, #304017, BioLegend) or 8 μg/ml of Alexa Fluor 488 conjugated anti-CD3 antibodies (clone OKT3, #16-0037-85, Invitrogen). The anti-CD3 antibodies were fluorescently labeled using an Alexa Fluor 488 antibody labeling kit (#A20181, Invitrogen). The cells were centrifuged for 2 min at 2,000 rpm and resuspended in 250 μl HBS buffer, labeled with the respective antibody on ice for 30 min and washed with HBS buffer. The cells were added to the SLBs 15 min prior to imaging.

### Supported Lipid Bilayers

Supported lipid bilayers were made using vesicle fusion and rupture on clean glass. In short, small unilamellar vesicles containing a mixture of 1-palmitoyl-2-oleoyl-*sn*-glycero-3-phosphocholine (POPC, Avanti Polar Lipids) and 1,2-dioleoyl-sn-glycero-3-[(N-(5-amino-1-carboxypentyl)iminodiacetic acid)succinyl] (DGS-NTA(Ni), #790404C; Avanti Polar Lipids) were made at different ratios. In addition, for one experiment a lipid mixture of 10 wt% of the anionic lipid 1,2-dioleoyl-*sn*-glycero-3-phospho-L-serine (DOPS, Avanti Lipids) and 90 wt% POPC was used. Glass cover slides (24 mm × 40 mm, thickness 1.5, Menzel-Gläzer) were cleaned for 30 min using a 80°C mixture of 75% sulfuric acid (99.9%, Sigma-Aldrich) and 25% hydrogen peroxide (30%, Sigma-Aldrich) and four press-to-seal silicon wells (4.5 mm in diameter, 1.6 mm in depth, Grace Bio-Labs) were attached to the clean glass slide. SLBs were formed by adding the different vesicle solutions in HBS buffer (0.5 mg lipids per ml) to each well and incubating for 1 h.

Rat CD2 molecules containing a double polyhistidine-tag (12xH) at the C-terminus and the human L3-12 TCR containing one polyhistidine-tag (6xH) each on the C-terminus of the α- and β-chain were made as described in Junghans et al. (2020) and were fluorescently labeled using an Alexa Fluor 647 antibody labeling kit (#A20186, Invitrogen). Depending on the desired ligand density, between 0.3 and 2.5 μg/ml of CD2 in HBS buffer was incubated with the SLB for 15–30 min before rinsing. The density of the CD2 molecules on the SLB was obtained from the intensity of single CD2 molecules on glass as described previously (Junghans et al., 2018, 2020). The successful formation of a mobile SLB was confirmed by fluorescence recovery after photobleaching before each experiment (Jönsson et al., 2008).

Blocking of the SLB was done with a 5% BSA solution (#A5611, Sigma-Aldrich) in HBS buffer, which was incubated with the SLB for 30 min before washing the sample. Stripping of the nickel ions from DGS-NTA(Ni) was performed with 100 mM ethylenediaminetetraacetic acid (EDTA) in HBS buffer. The EDTA solution was incubated with the SLB for 10 min before washing, a procedure that was repeated twice before adding the Jurkat T cells. In all the washing experiments the cells were added to the SLB 15 min before washing with HBS buffer.

For a subset of experiments, the Ni^2+^ ions in DGS-NTA(Ni) were replaced with either Cu^2+^ or Co^2+^ ions. A 5 wt% DGS-NTA(Ni) SLB was first washed with 100 mM EDTA as described above. The SLB was then reloaded with metal ions by incubating the SLB with a 5 mM solution of either (i) CoCl_2_ (Sigma-Aldrich), (ii) NiCl_2_ (Sigma-Aldrich), or (iii) CuCl_2_ (Sigma-Aldrich) in HBS, adjusted to a pH of 7.4 with tris(hydroxymethyl)aminomethane (TRIS, Sigma-Aldrich), for 40 min before rinsing again with HBS. It was verified using fluorescence recovery after photobleaching that all the different DGS-NTA complexes bound polyhistidine-tagged CD2.

### Imaging

A customized inverted Nikon Eclipse Ti microscope with a motorized stage was used for imaging of the samples using either total internal reflection fluorescence (TIRF) or epi-fluorescence microscopy. An Oxxius LBX diode laser operating at 488 nm was used to monitor the Fluo4-AM signal inside the cells as well as the anti-CD45 and anti-CD3 antibodies at the cell surface. An Oxxius LBX diode laser operating at 638 nm was used to monitor CD2 on the SLB. The images were acquired on a Photometrics Prime 95B sCMOS camera.

Cell-SLB contacts were monitored with an 100x oil immersion objective (NA 1.49, Nikon Corporation) in TIRF mode, whereas to obtain signaling fractions of triggered Jurkat T cells, the cells, containing Fluo4-AM, were visualized with a 10x air objective (Nikon Corporation) in epi-fluorescence mode. To capture the increase in Fluo4-AM intensity upon binding of Ca^2+^ ions a 150 frame long time-lapse video with a time between frames of 5 or 6 s was started at the time of cell addition to the sample. The motorized stage was used to measure four samples in each experiment. In each experiment, one of the four surfaces was coated with the anti-CD3 antibody OKT3 (clone OKT3, #16-0037-85, Invitrogen) at a concentration of 10 μg/mL for 60 min as a positive control to confirm the ability of the cells to be activated. All images were acquired with an exposure time of 100 ms via μManager version 1.4 (Edelstein et al., 2010).

### Image Analysis

Calcium signaling from the bound cells was analyzed using a custom-written MATLAB script (R2020a, MathWorks). Only the first 150 cells binding to the surface were used in the analysis, which were detected using the script pkfnd in MATLAB (Blair and Dufresne, 2020; Supplementary Figure 1). Cells that signaled directly on landing or were present already at frame 1 were removed from the analysis and were not included in the 150 cells that were analyzed. The intensity for each cell was plotted as a function of time. When the intensity increased above a user-set threshold value and remained above this value the cell was considered to have bound to the surface. When the intensity increased more than 2.5 times above the baseline, non-activated intensity of the bound cell, the cell was considered to have signaled (Supplementary Figure 1). The intensity vs. time plot for each cell was saved and inspected manually to verify that the script had classified the cell correctly (Supplementary Figures 2, 3). All images had the background intensity subtracted using a rolling ball radius of 50 pixels in ImageJ (1.53e) (Schneider et al., 2012).

The accumulation of CD2 in the cell contacts was detected using a customized MATLAB script as described in detail elsewhere (Jönsson et al., 2016). In short, the outline of the cell-SLB contact was created by thresholding the intensity of CD2 in the SLB to detect regions of CD2 accumulation. The average intensity in each contact was saved yielding the sum of bound and free ligands in the contact. The intensity outside the contact gave the free ligand density, which was corrected for ligand exclusion in the cell-SLB contact by 25% (Junghans et al., 2020). The intensities were converted to protein densities using the single molecule intensity from one protein as described by Junghans et al. (2018, 2020).

A line profile of the intensity through each of the cell-SLB contacts was used to analyze the anti-CD45 antibody distribution. The exclusion of CD45 from the contact was determined as 1-*I*_in_/*I*_out_, where *I*_in_ is the intensity inside the contact and *I*_out_ is the maximum intensity of the anti-CD45 signal in the area outside the contact.

## Results

### Cell Signaling on Ligand-Free SLBs

It was first assessed at what concentration of DGS-NTA(Ni) in the SLB the cells attach. Jurkat T cells on SLBs without DGS-NTA(Ni) could be washed off after being added to the SLB (Supplementary Figure 4). When having 1 wt% DGS-NTA(Ni) approximately half of the cells remained after washing, which increased to 90% and higher for SLBs with ≥2 wt% DGS-NTA(Ni) (Supplementary Figure 4). When blocking the SLBs with 5% BSA before adding the cells, the majority of cells could be washed off (Supplementary Figure 4). The same was true when washing the SLBs with EDTA that strips the nickel ions from the DGS-NTA(Ni) (Supplementary Figure 4). In addition, replacing the net negatively charged DGS-NTA(Ni) with 10 wt% of the anionic lipid DOPS did not result in cell attachment (Supplementary Figure 4), altogether indicating that it is the nickel ions in DGS-NTA(Ni) that are responsible for the cell attachment. The cell attachment was furthermore not restricted to Jurkat T cells since the human monocytic cell line THP-1 also bound to DGS-NTA(Ni) SLBs, and the cells could be detached after washing with EDTA (Supplementary Figure 4).

**FIGURE 1:**
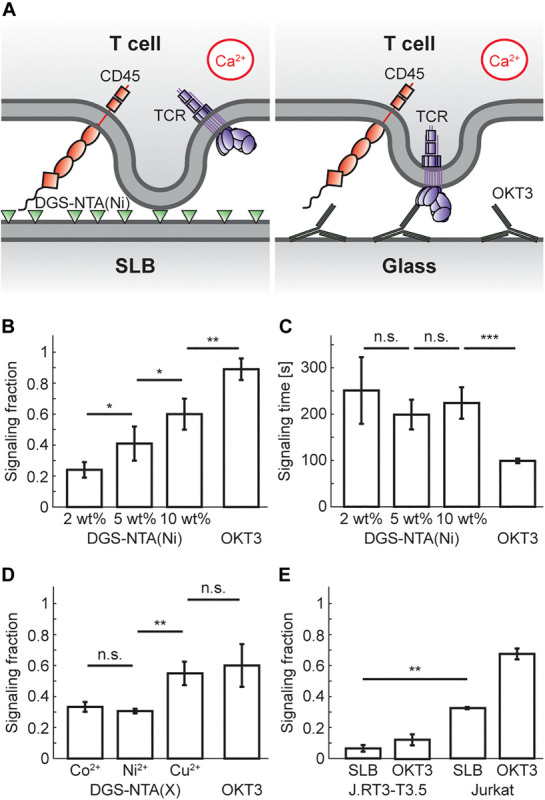
T-cell signaling fraction increases with concentration of DGS-NTA(Ni) in the SLB. **(A)** Schematic illustration showing calcium signaling by either (*left*) DGS-NTA(Ni) in an SLB or (*right*) anti-CD3 antibodies on glass. **(B)** The fraction of signaling T cells for either ligand-free SLBs with DGS-NTA(Ni) or OKT3 on a glass slide (mean signaling fraction ± s.d.). **(C)** The time between cell landing and calcium signaling for either ligand-free SLBs with DGS-NTA(Ni) or OKT3 on a glass slide (mean signaling time ± s.d.). **(D)** The signaling fraction binding to a ligand-free SLB containing 5 wt% DGS-NTA(X), with X being either Co^2+^, Ni^2+^ or Cu^2+^. **(E)** The signaling fraction of J.RT3-T3.5 cells (*left*) and Jurkat T cells (*right*) on SLBs with ligand-free 5 wt% DGS-NTA(Ni) and on OKT3-coated glass. Data are shown for three to five experiments, except for E which is from two experiments, with 150 cells in each experiment. A two-sample *t*-test in MATLAB was used for statistical analysis with: * <0.05, ** <0.01, *** <0.001.

Having established that SLBs containing ≥2 wt% DGS-NTA(Ni) bind T cells, we investigated whether they also induces calcium signaling in the cells (Figure 1A). It was found that all SLBs induced calcium signaling, but to various extents. The fraction of signaling cells increased with the concentration of DGS-NTA(Ni) in the SLB and was 24 ± 5% (mean ± s.d.), 41 ± 11%, and 60 ± 10% at 2, 5, and 10 wt% DGS-NTA(Ni), respectively (Figure 1B and Supplementary Movie 1–3). This is similar to what was previously observed by Ponjavic et al. (2018) who observed a signaling fraction of around 50% for a ligand-free SLB containing 5% DGS-NTA(Ni). The fraction of cells showing calcium signaling on glass coated with the anti-CD3 antibody OKT3 was higher with 89 ± 7% (Figure 1B and Supplementary Movie 4). Whereas the signaling fraction increased with the amount of DGS-NTA(Ni) in the SLB no significant trend in the time between cell landing and signaling was observed between the different SLBs (Figure 1C). There was a higher spread in the signaling times for cells on SLBs compared to cells binding to OKT3-coated glass, the latter also having an average signaling time that was more than twice as fast as that of the SLBs.

In addition, SLBs containing 5 wt% DGS-NTA chelated with either (i) Co^2+^, (ii) Ni^2+^, or (iii) Cu^2+^ were also made. All three DGS-NTA complexes showed significant signaling, with DGS-NTA(Cu) signaling significantly more than the two other complexes (Figure 1D). This is in agreement with the protein affinity for Cu^2+^ being the highest of these three metal ions in immobilized metal ion affinity chromatography (Porath, 1992), illustrating that other factors than just the positive charge of the nickel ions in DGS-NTA(Ni) influences calcium signaling. It is possible that DGS-NTA(Ni) could induce calcium signaling by another mechanism than TCR triggering, for example by acting on mechanosensitive calcium channels in the plasma membrane (Pottosin et al., 2015). To investigate this, calcium signaling experiments were performed on a 5 wt% DGS-NTA(Ni) SLB with the Jurkat T cell mutant J.RT3-T3.5 that lacks the beta chain of the TCR. The J.RT3-T3.5 cells showed considerably lower signaling than Jurkat T cells with 7 ± 2% (mean ± s.d., *n* = 2) of the J.RT3-T3.5 cells signaling on 5 wt% DGS-NTA (Figure 1E), indicating that DGS-NTA(Ni) signaling is mainly via TCR triggering.

### DGS-NTA(Ni) Dominates Signaling Also With Ligands in the SLB

**FIGURE 2:**
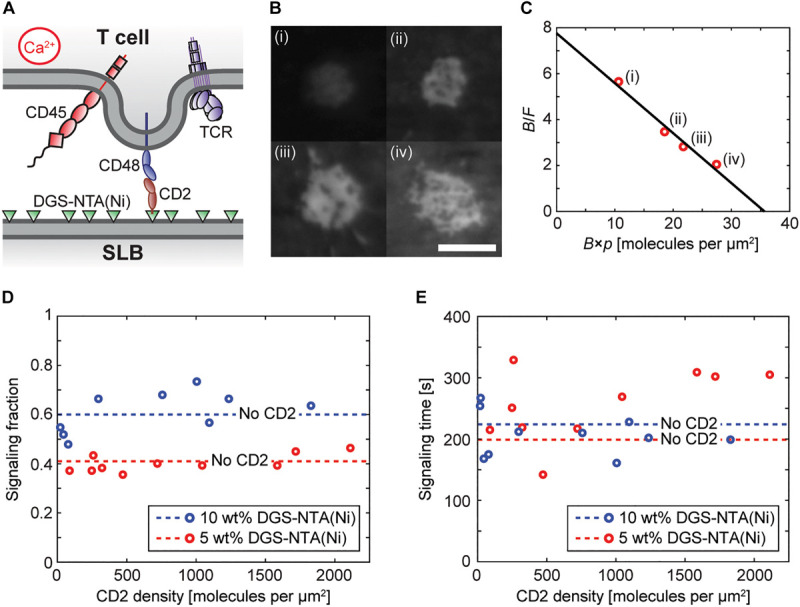
DGS-NTA(Ni) dominates over ligand binding in inducing calcium signaling. **(A)** An illustration of a Jurkat T cell interacting with a rat CD2-functionalized SLB to induce calcium signaling. **(B)** Fluorescence images of accumulated CD2 in four different cell-SLB contacts with varying free (bound) CD2 density: (i) 68 (381) CD2 molecules per μm^2^, (ii) 189 (659) CD2 molecules per μm^2^, (iii) 249 (697) CD2 molecules per μm^2^, and (iv) 361 (739) CD2 molecules per μm^2^. The average cell-SLB contact area increased from 19 to 26 μm^2^ between (i) and (iv). The scale bar is 5 μm and the scale is the same for all images. **(C)** Zhu-Golan plot for the data in (B) (red circles) with: *F*, free CD2 density; *B*, bound CD2 density; and *p*, the fraction of the cell surface area within the cell-SLB contact. The solid line is a linear fit to the data. **(D,E)** The signaling fraction and average signaling time at different densities of CD2 on SLBs containing 5 wt% DGS-NTA(Ni) (red circles) and 10 wt% DGS-NTA(Ni) (blue circles). Each data point represents one experiment with 150 analyzed cells. The dashed lines represent the average values on a ligand-free SLB [5 wt% DGS-NTA(Ni): blue dashed line, 10 wt% DGS-NTA(Ni): red dashed line].

It was shown in the previous section that DGS-NTA(Ni)-containing SLBs alone can induce T-cell signaling and that the fraction of cells that signal increases with the amount of DGS-NTA(Ni) in the SLB. However, SLBs will in general be functionalized with protein ligands, which might be able to shield and reduce interactions between the DGS-NTA(Ni) and the cell. To investigate the influence of ligands on calcium signaling by DGS-NTA(Ni) we next investigated how the fraction of signaling Jurkat T cells, transfected with the non-signaling receptor CD48, depends on the amount of the ligand CD2 in the SLB (Figure 2A). SLBs containing up to 2,000 CD2 molecules per μm^2^ were made and the ligands were observed to bind to CD48 in the contacting T cells (Figure 2B). The relative accumulation of CD2 in the cell-SLB contacts could also be presented in a Zhu-Golan plot (Figure 2C). The slope of the Zhu-Golan plot corresponds to −1/*K*_d_ of the CD2-CD48 interaction, where *K*_d_ is the two-dimensional dissociation coefficient, whereas the intersect with the *x*-axis gives the density of mobile CD48 receptors on the cell (Zhu et al., 2007). A linear fit to the data in Figure 2C gave a *K*_d_ of 5 molecules per μm^2^, which agrees with what has previously been measured for this interaction (Junghans et al., 2020). From the *x*-intersect a density of mobile CD48 receptors of 36 molecules per μm^2^ was obtained. Assuming a cell area of 700 μm^2^ and a fraction of mobile CD48 receptors of 60% (Junghans et al., 2020) this gave a total number of 42,000 CD48 molecules per cell. This is comparable to an average number of 47,000 CD48 molecules per cell measured using flow cytometry (Supplementary Figure 5).

The concentration of CD2 in the SLB did not change the fraction of signaling cells compared to ligand-free SLBs (Figure 2D and Supplementary Movie 5,6). Thus, SLBs with similar amounts of CD2 did signal approximately 50% more on SLBs containing 10 wt% DGS-NTA(Ni) compared to SLBs containing 5 wt% DGS-NTA(Ni). There was also no statistical difference for the signaling time with and without ligand, accessed by a two-sample *t*-test, although there was a larger spread in this data (Figure 2E). At a density of 2,000 CD2 molecules per μm^2^ the average distance between two CD2 molecules is 22 nm, which appears to be enough for the cell surface to be able to interact with DGS-NTA(Ni) in the SLB. It is also worth stressing that there are of the order of 100,000 DGS-NTA(Ni) molecules per μm^2^ in a 10 wt% DGS-NTA(Ni) SLB, and the number of available DGS-NTA(Ni) molecules in the SLB is thus negligibly affected by CD2 binding under these conditions.

### BSA Blocking Reduces Calcium Signaling Without Affecting CD45 Exclusion

**FIGURE 3:**
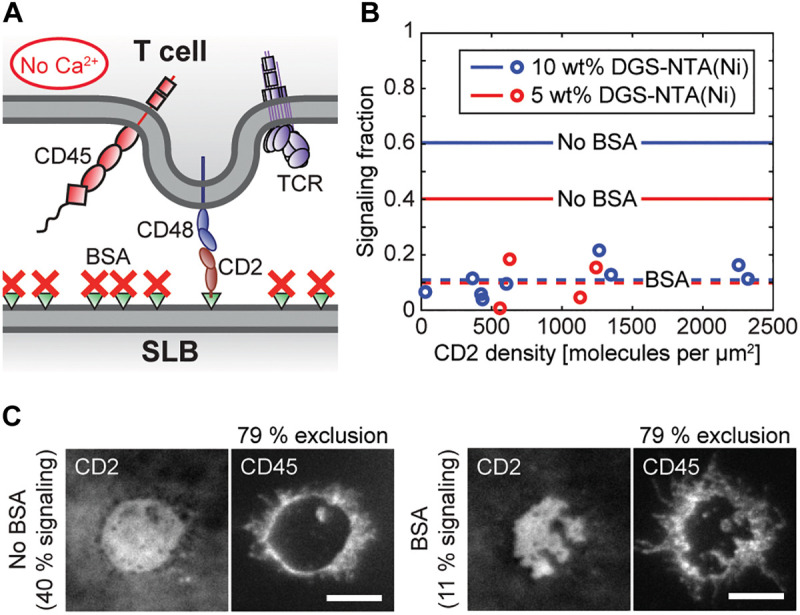
Calcium signaling is significantly reduced upon blocking the SLB with BSA, without affecting CD45 exclusion. **(A)** Schematic illustration of the BSA-blocked and CD2-functionalized SLB in contact with a T cell. **(B)** The signaling fraction of cells on BSA-blocked SLBs containing either 5 wt% DGS-NTA(Ni) (red circles) or 10 wt% DGS-NTA(Ni) (blue circles) at different CD2 densities. Each data point represents one experiment with 150 analyzed cells. The solid lines represent the average values on a non-blocked SLB [5 wt% DGS-NTA(Ni): blue line, 10 wt% DGS-NTA(Ni): red line], whereas the dashed lines correspond to the average signaling fraction for the BSA-blocked SLBs. **(C)** Fluorescence images of two representative cell-SLB contacts in the CD2 and the CD45 channel without (*left*) and with (*right*) BSA blocking. The SLBs contained 5 wt% DGS-NTA(Ni) and had ∼1,000 CD2 molecules per μm^2^. The scale bars are 5 μm.

When blocking the CD2-functionalized SLBs with BSA before adding the cells (Figure 3A) the majority of cells still bound to the SLB, but, the signaling cell fraction decreased to on average 11% (Figure 3B and Supplementary Movie 7). This was not dependent on whether the SLB contained 5 or 10 wt% DGS-NTA(Ni). There appeared to be a weak increase in signaling fraction with CD2 density, but, the correlation between signaling fraction and CD2 density in Figure 3B was not statistically significant. A reduction of signaling to ∼10% was also observed by Ponjavic et al. (2018) when blocking a ligand-free SLB containing 5% DGS-NTA(Ni) with BSA solution, which could indicate that this value is approximately the background signaling level for these cells and that the signaling fraction above this value for the blocked SLBs is due to ligand-independent triggering caused by CD2 binding CD48.

Since the exclusion of the phosphatase CD45 from cell-SLB contacts has previously been shown to be a key event in inducing T-cell signaling (James and Vale, 2012; Cordoba et al., 2013; Chang et al., 2016) we labeled the cells with fluorescently labeled antibodies against CD45 to study the distribution of CD45 in- and outside the cell-SLB contacts. Similar to previous observations we found that CD45 was excluded from cell-SLB contacts created by CD2-CD48 binding (Chang et al., 2016; Bakalar et al., 2018; Fernandes et al., 2019; Figure 3C). Although the signaling fraction of cells varied significantly between SLBs with and without BSA the exclusion level of CD45 was 79% in both cases [±5% (6%) s.d., *n* = 32 (47) for a 5 wt% DGS-NTA(Ni) SLB containing ∼1,000 CD2 molecules per μm^2^ with (without) BSA]. In contrast, antibodies against the TCR complex showed no clear exclusion in the cell-SLB contact (Supplementary Figure 6). Although the size of the antibody can potentially influence the distribution of CD45 in the contact, the found exclusion levels are nonetheless comparable to the CD45 exclusion that has previously been observed for other systems having CD2 binding CD48 on T cells (Chang et al., 2016; Fernandes et al., 2019).

## Discussion

We demonstrate how nickel-chelating lipids in an SLB can induce calcium signaling in T cells even when having protein ligands on the SLB. The fraction of cells that signaled increased with the concentration of nickel-chelating lipids and reached 60% for an SLB with 10 wt% DGS-NTA(Ni), independently of whether the SLB was functionalized with protein ligands or not. This was lower than the ∼90% signaling fraction we obtained for the same cells when using glass coated with the anti-CD3 antibody OKT3, but still of the same magnitude as observed in other studies where non-TCR ligands were responsible for inducing calcium signaling (Chang et al., 2016; Ponjavic et al., 2018). When blocking the SLB with BSA the signaling fraction decreased to 11%, indicating that ligand binding only weakly, if at all, induces calcium signaling for the current system and conditions. A reduction in signaling by nickel-chelating lipids is expected by using other blocking molecules as well, for example including polyethylene glycosylated lipids in the SLB or having higher levels of non-binding ligands. The difference in signaling fraction does not appear to be due to different levels of CD45 exclusion in the various systems, since both BSA blocked and unblocked SLBs containing CD2 showed a similar level of exclusion of CD45. This indicates that the calcium signaling induced by DGS-NTA(Ni) is caused by a different mechanism than in ligand-independent triggering. However, DGS-NTA(Ni) appears to induce signaling via TCR triggering since the fraction of signaling cells decreased significantly when using TCR-deficient J.RT3-T3.5 cells instead of Jurkat T cells. A similar signaling fraction and signaling time, both with and without BSA blocking (Supplementary Movies 8, 9), was furthermore observed when instead of CD2 having L3-12 TCR in the SLB, which bound to non-signaling HLA-DQ8-glia-α1 on the Jurkat T cell, and which was expressed at considerably higher levels than that of CD48 (Junghans et al., 2020).

To what extent DGS-NTA(Ni) influences calcium signaling will likely depend on both the state of the cell, its T-cell type and the experimental conditions, and for this reason it is mainly the relative changes that are of general interest. We can nevertheless say that for our system signaling via binding of the TCRs directly to antibodies on glass was the most potent, followed by DGS-NTA(Ni) and then lastly CD45 exclusion in close contacts according to ligand-independent triggering. That ligand-independent triggering caused by CD2-CD48 binding is not clearly observable in this study is in contrast to previous studies where it has been shown that creating close contacts that exclude CD45 to a similar degree as found here will trigger the TCR and induce calcium signaling for a majority of the bound cells (Chang et al., 2016; Fernandes et al., 2019). One reason for this could be that the cells used in this study express lower amounts of TCR (on average ∼4,000 TCRs per cell with the flow cytometry peak corresponding to ∼8,000 TCRs per cell; Supplementary Figure 5) compared to the Jurkat T cells and primary CD4+ T cells used in these studies (12,000–14,000 TCRs per cell for the Jurkat T cells and ∼40,000 TCRs per cell for the primary CD4+ T cells; (Fernandes et al., 2019) and personal communication with Ricardo A. Fernandes). It was found in Fernandes et al. (2019) that an average of 16 TCRs were residing in the close contact zones upon calcium signaling indicating that calcium signaling depends on multiple TCRs being triggered in close proximity. That triggering of multiple TCRs in close proximity is connected to T-cell activation has also been demonstrated in other studies (Manz et al., 2011; Lin et al., 2019). It is therefore possible that the lower density of TCR for the cells in this study is not sufficient to give rise to enough TCR triggering events to initiate calcium signaling by ligand-independent triggering alone. It should also be noted that in Chang et al. (2016), the dependence of bilayer contact on CD2-CD48 binding *per se* was controlled for by showing that cells lacking CD48 did not settle on CD2-presenting bilayers. This indicates that the interaction between the cells and DGS-NTA(Ni) was weaker for these cells than what is observed in this study for similar levels of DGS-NTA(Ni) in the SLB. Why signaling takes place when T cells bind to OKT3-coated glass or to SLBs with DGS-NTA(Ni) in this study can only be speculated about. However, one possibility is that both these systems engage the TCR to some extent, which via conformational changes could make the TCR more sensitive to signaling (Mariuzza et al., 2020). It could also be, in line with the low amount of TCR on the cells, that binding the TCR locally increases the concentration of triggered TCRs above the threshold needed to induce calcium signaling.

In summary, we show that DGS-NTA(Ni) can have a significant effect on binding T cells and induce calcium signaling even when having ligands in the SLB. The molecular mechanism by which this happens can only be speculated about but appears not to be due to increasing the level of CD45 exclusion, although this is likely one crucial part for signaling on the ligand-free SLBs. It is instead possible that the charged DGS-NTA(Ni) acts as a weak ligand for the TCR which can induce calcium signaling unless the nickel-chelating lipids are blocked. The amount of calcium signaling induced by DGS-NTA(Ni) vs. ligand-independent triggering can vary between different cells and conditions. However, it should generally be of importance to keep the nickel-chelating lipids in the SLB blocked in order to ensure minimum influence on the cells and to keep them in a resting state when binding to protein ligands on the SLB.

## Data Availability Statement

The raw data supporting the conclusions of this article are available upon request from the corresponding author, without undue reservation.

## Author Contributions

TD, VJ, PJ, and JH: conceptualization. TD and MC: formal analysis. TD: investigation. PJ and VJ: resources and supervision. TD and PJ: writing – original draft and visualization. TD, PJ, and VJ: writing – review and editing. PJ: project administration and funding acquisition. All authors contributed to the article and approved the submitted version.

## Conflict of Interest

The authors declare that the research was conducted in the absence of any commercial or financial relationships that could be construed as a potential conflict of interest.
