# Supplementary material for: Calcium Signaling in T Cells Is Induced by Binding to Nickel-Chelating Lipids in Supported Lipid Bilayers
Source: Front Physiol. 2021 Jan 21;11:613367. doi: 10.3389/fphys.2020.613367 (PMC7859345; doi:10.3389/fphys.2020.613367)
Supplement: Supplementary file 1 [file Data_Sheet_1.pdf]

Supplementary Material

Calcium Signaling in T Cells Is Induced by Binding to Nickel-Chelating Lipids in Supported Lipid Bilayers

Tommy Dam[1], Victoria Junghans[1], Jane Humphrey[2], Manto Chouliara[1], Peter Jönsson[1]

[1]Department of Chemistry, Lund University, Lund, Sweden
[2]Department of Chemistry, University of Cambridge, Cambridge, UK

*** Correspondence:**
Corresponding Author
peter.jonsson@fkem1.lu.se

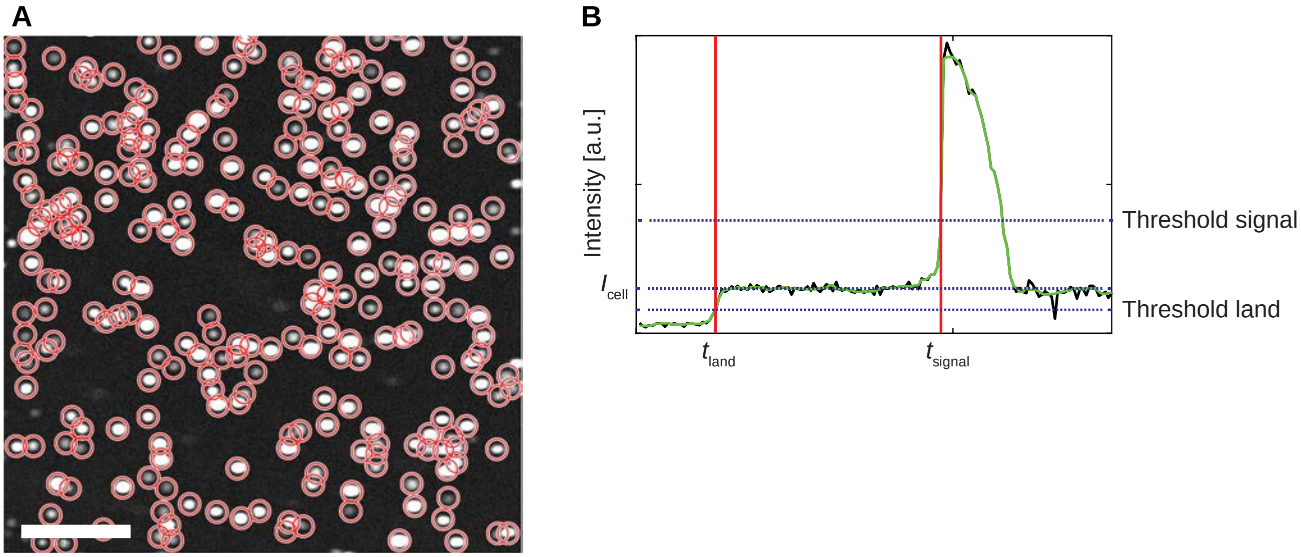

Supplementary Figure 1. The signaling fraction and signaling time was determined using a custom-written MATLAB script. **(A)** Image showing bound and detected cells. Each red circle corresponds to one detected cell. The scale bar is 100 µm. **(B)** Intensity profile from one detected cell in A at different image frames (black line: raw data, green line: moving median-filtered data). The cell landed at t_{land} and signaled at t_{signal}, defined by the intensity increasing above “Threshold land” and “Threshold signal”, respectively. The first of these thresholds is user set, whereas the second is given by $2.5 \times I_{cell}$, where I_{cell} is the intensity of the non-signaling cell.

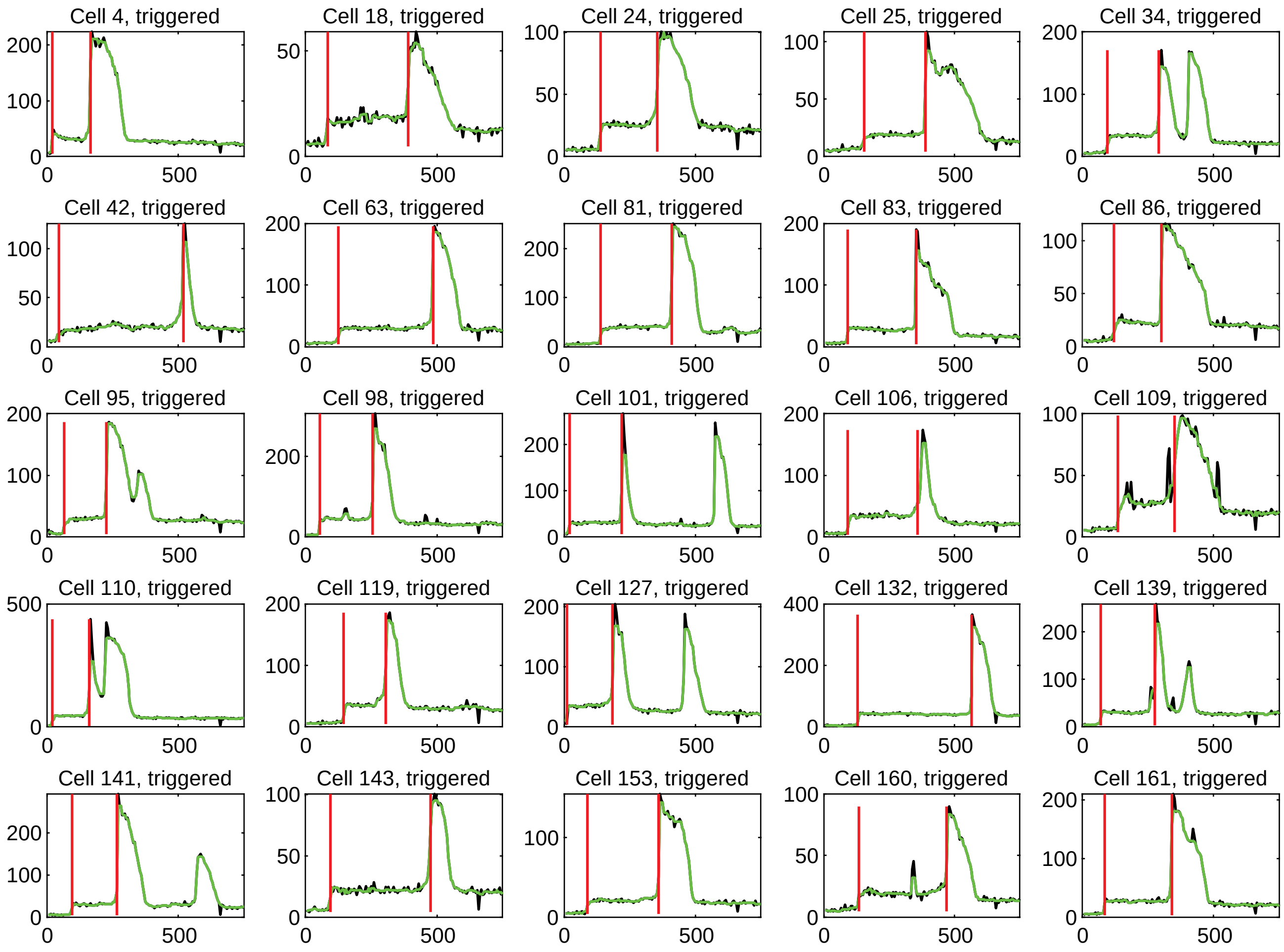

Supplementary Figure 2. Examples of intensity traces for different signaling cells (black line: raw data, green line: moving median-filtered data). The first red line corresponds to the time when the cell lands and the second red line the time when the cell signals. The *y*-axis shows the intensity in arbitrary units and the *x*-axis the time in seconds.

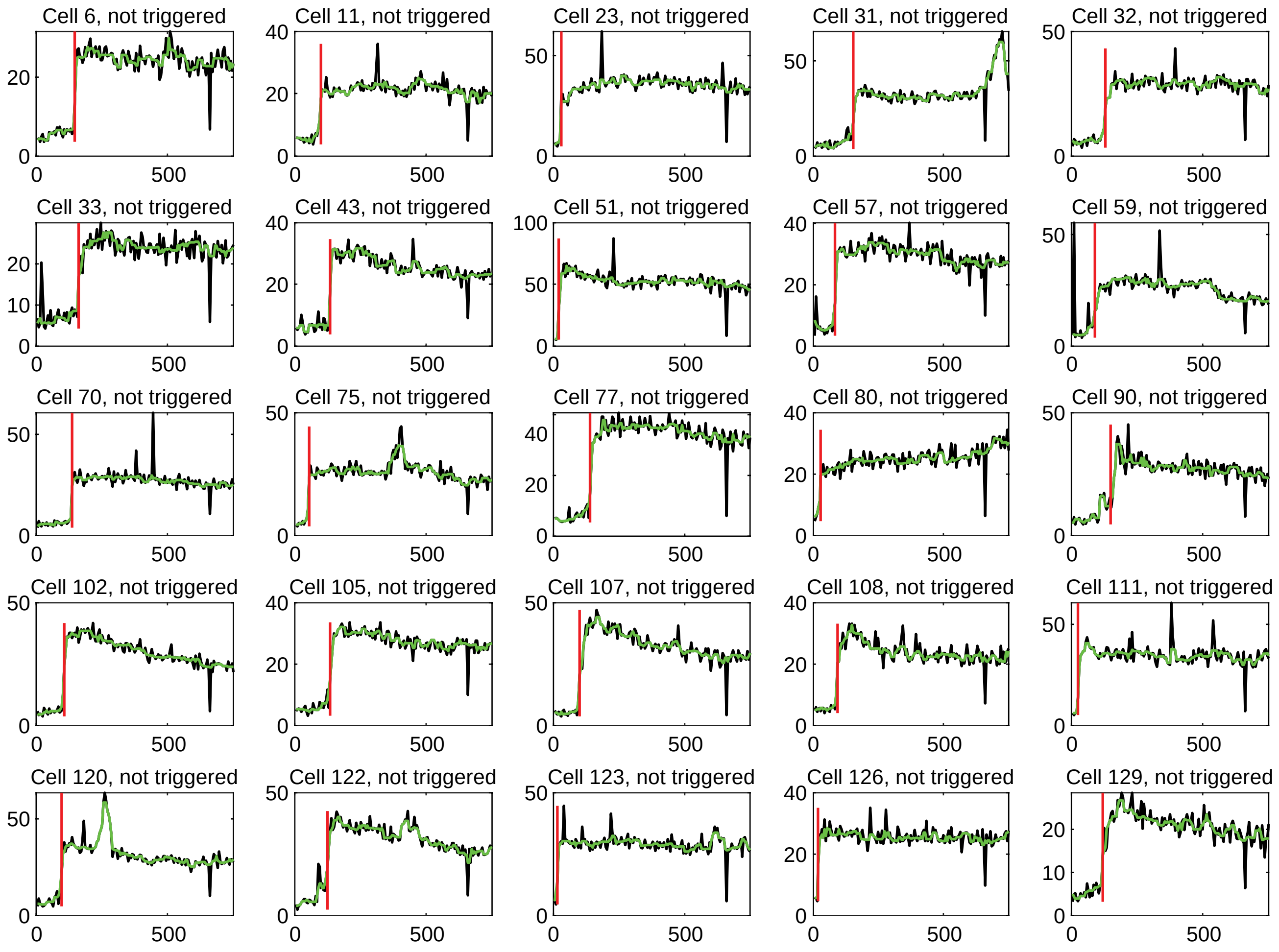

Supplementary Figure 3. Examples of intensity traces for different non-signaling cells (black line: raw data, green line: moving median-filtered data). The red line corresponds to the time when the cell lands. The *y*-axis shows the intensity in arbitrary units and the *x*-axis the time in seconds.

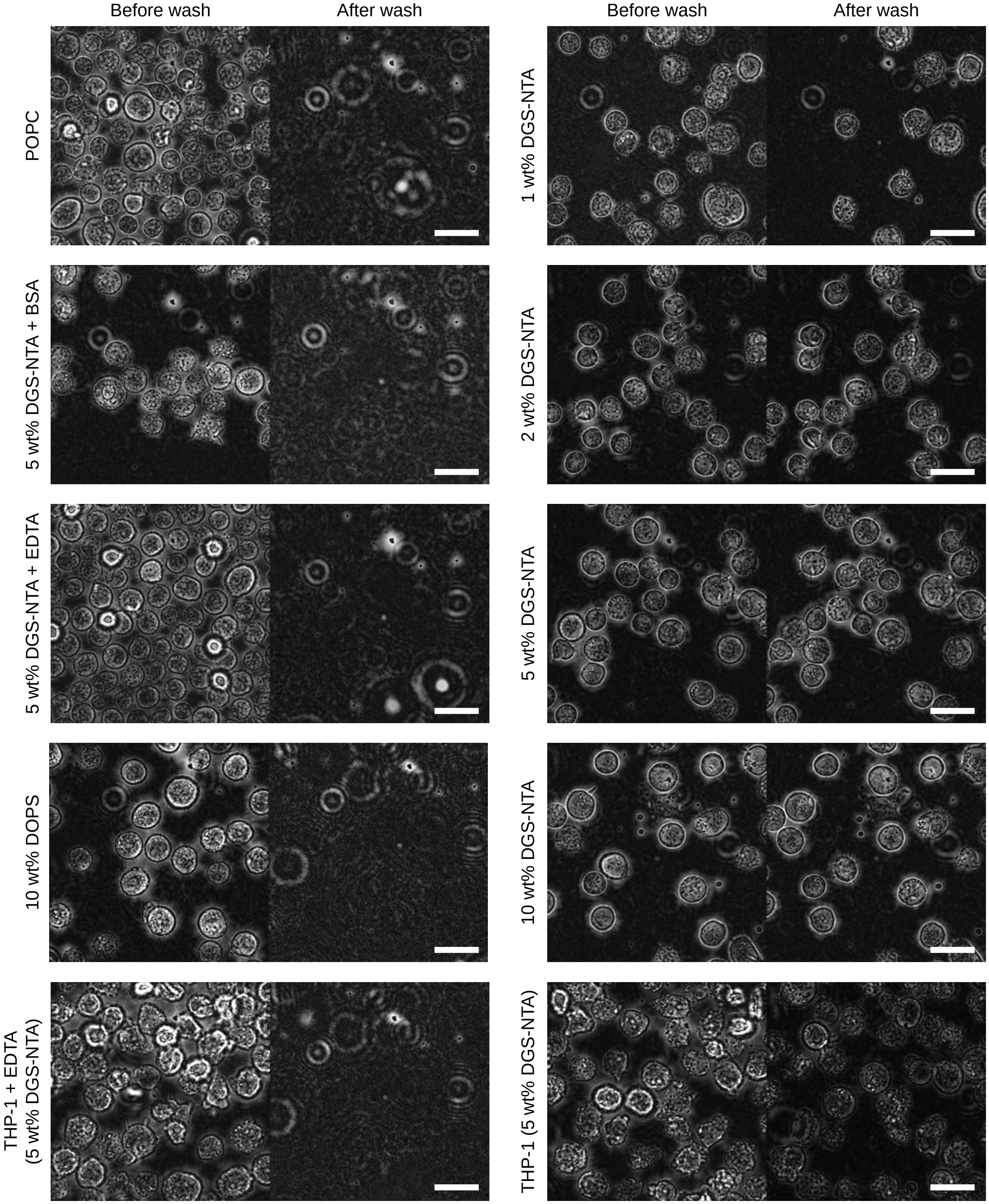

Supplementary Figure 4. Cells attach to SLBs containing ≥2 wt% DGS-NTA. The figures show bright field images before (*left*) and after (*right*) washing for different SLB systems. All cells are Jurkat T cells except the lowest row which are THP-1 cells. The scale bar is 20 μm in all images.

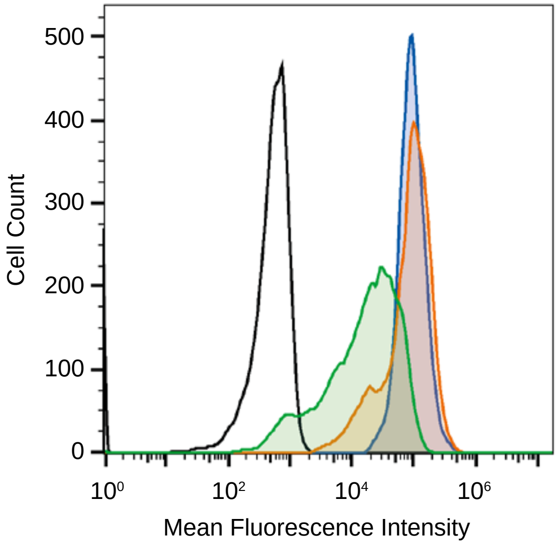

Supplementary Figure 5. Cell surface expression of CD3, CD48 and CD45. Mean fluorescence intensity of the isotype PE α-mouse IgG1 (black line), PE α-human CD3 (green line), PE α-rat CD48 (orange line) and PE α-human CD45 (blue line). Quantibrite analysis was used to convert the intensity to an average number of molecules per cell: CD3 = 7 700 molecules per cell (TCR = CD3/2 = 3 900 molecules per cell), CD45 = 59 000 molecules per cell and CD48 = 47 000 molecules per cell.

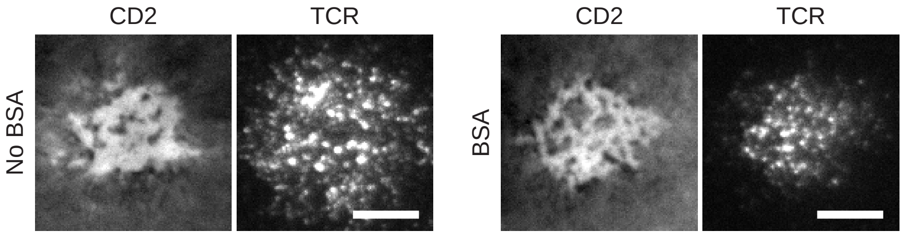

Supplementary Figure 6. Fluorescence images of representative cell-SLB contacts in the CD2 and the TCR channel without (*left*) and with (*right*) BSA blocking. The SLBs contained 10 wt% DGS-NTA(Ni) and had ~700 CD2 molecules per μm^2. The scale bar is 5 μm.

Supplementary Movie Legends

Supplementary Movie 1. Fluo4-AM fluorescence signal for Jurkat T cells binding to a ligand-free SLB containing 2 wt% DGS-NTA(Ni). The entire movie is 750 seconds long and the scale bar is 50 μm.

Supplementary Movie 2. Fluo4-AM fluorescence signal for Jurkat T cells binding to a ligand-free SLB containing 5 wt% DGS-NTA(Ni). The entire movie is 750 seconds long and the scale bar is 50 μm.

Supplementary Movie 3. Fluo4-AM fluorescence signal for Jurkat T cells binding to a ligand-free SLB containing 10 wt% DGS-NTA(Ni). The entire movie is 750 seconds long and the scale bar is 50 μm.

Supplementary Movie 4. Fluo4-AM fluorescence signal for Jurkat T cells binding to an OKT3-coated glass slide. The entire movie is 750 seconds long and the scale bar is 50 μm.

Supplementary Movie 5. Fluo4-AM fluorescence signal for Jurkat T cells binding to a 5 wt% DGS-NTA(Ni) SLB functionalized with 322 CD2 molecules per μm^2. The entire movie is 750 seconds long and the scale bar is 50 μm.

Supplementary Movie 6. Fluo4-AM fluorescence signal for Jurkat T cells binding to a 10 wt% DGS-NTA(Ni) SLB functionalized with 1236 CD2 molecules per μm^2. The entire movie is 750 seconds long and the scale bar is 50 μm.

Supplementary Movie 7. Fluo4-AM fluorescence signal for Jurkat T cells binding to a BSA-blocked SLB containing 10 wt% DGS-NTA(Ni) and being functionalized with 2321 CD2 molecules per μm^2. The entire movie is 750 seconds long and the scale bar is 50 μm.

Supplementary Movie 8. Fluo4-AM fluorescence signal for Jurkat T cells binding to a 10 wt% DGS-NTA(Ni) SLB functionalized with 629 L3-12 TCR molecules per μm^2. The signaling fraction of cells was determined to 0.52 and the average signaling time to 220 s. The entire movie is 750 seconds long and the scale bar is 50 μm.

Supplementary Movie 9. Fluo4-AM fluorescence signal for Jurkat T cells binding to a BSA-blocked SLB containing 10 wt% DGS-NTA(Ni) and being functionalized with 1930 L3-12 TCR molecules per μm^2. The signaling fraction of cells was determined to 0.05 and the average signaling time to 250 s. The entire movie is 750 seconds long and the scale bar is 50 μm.
